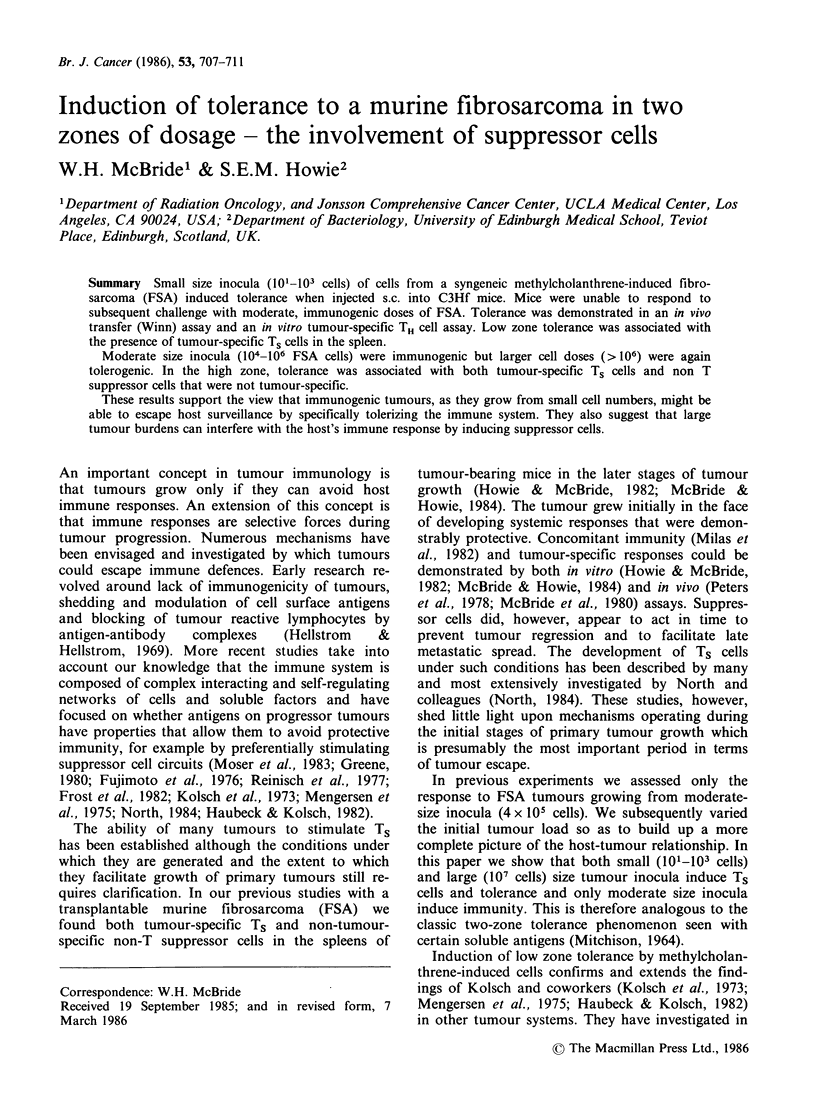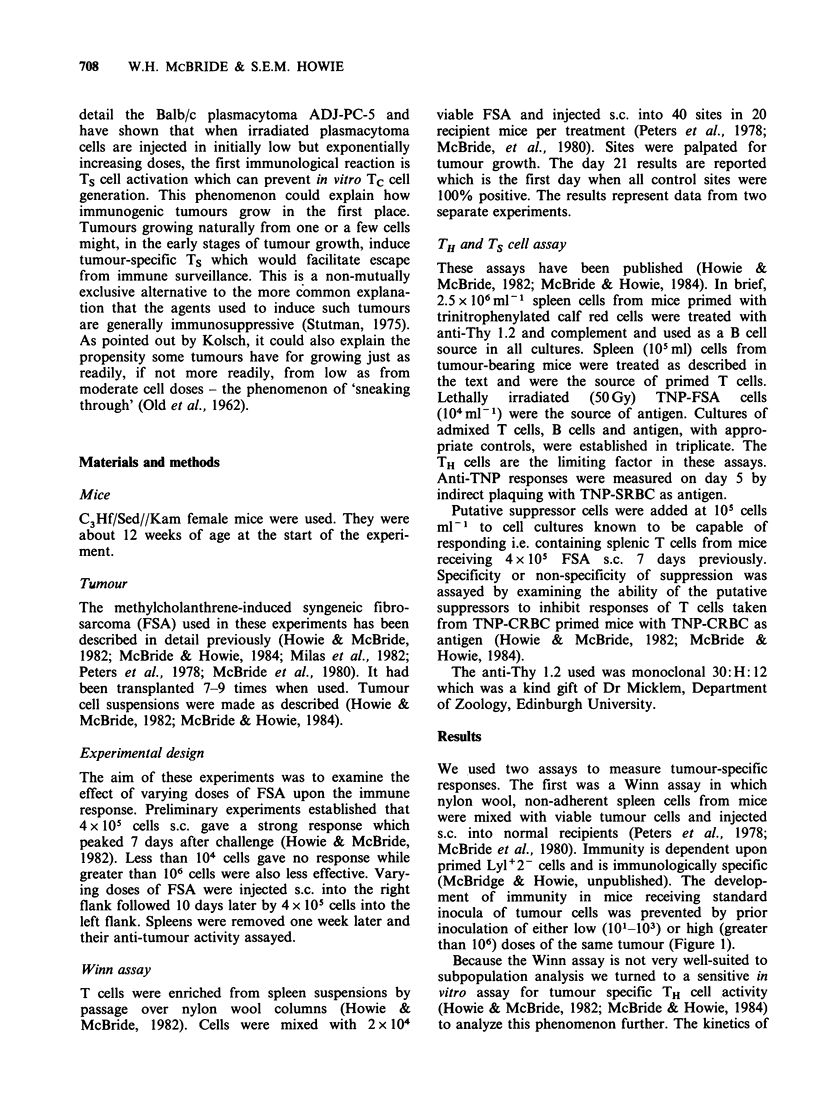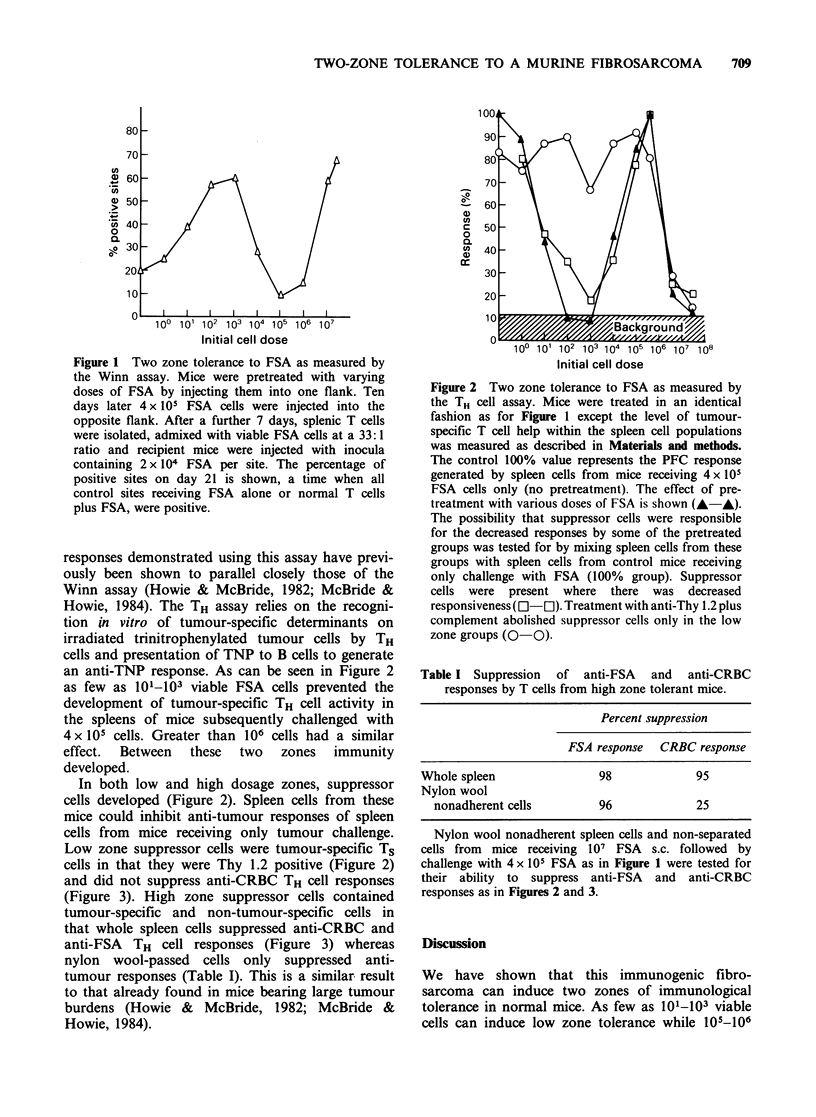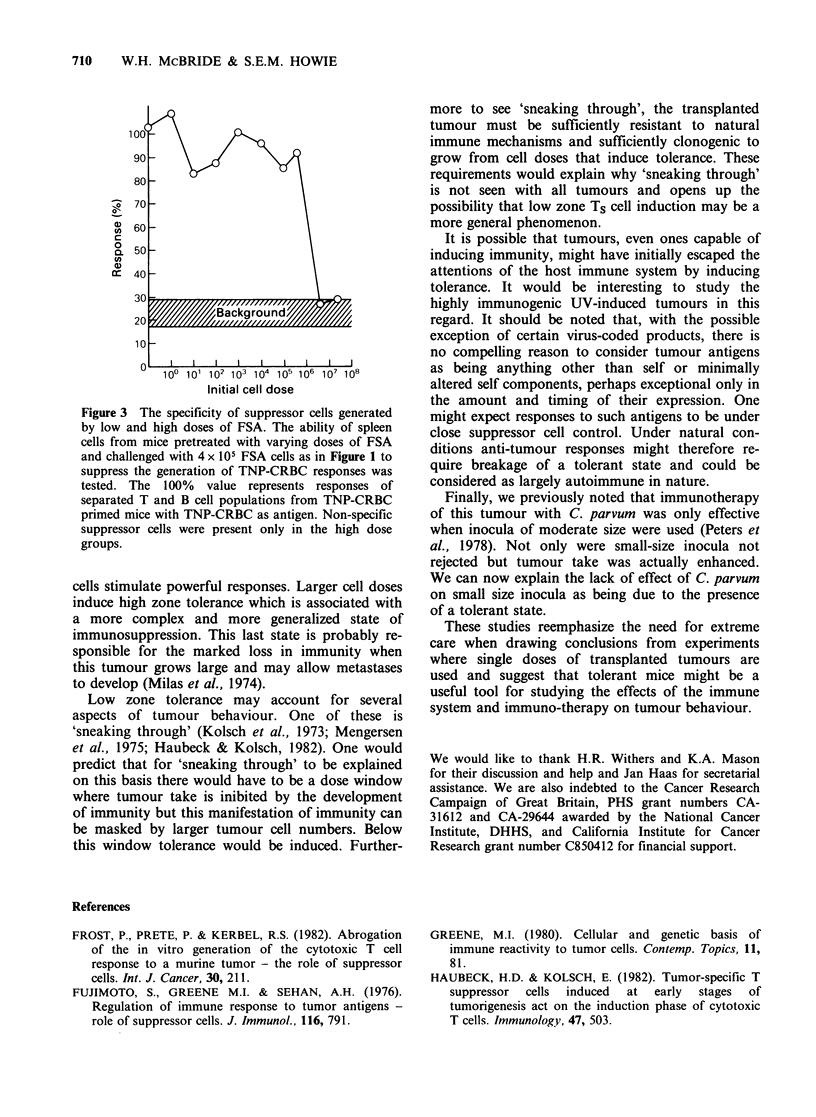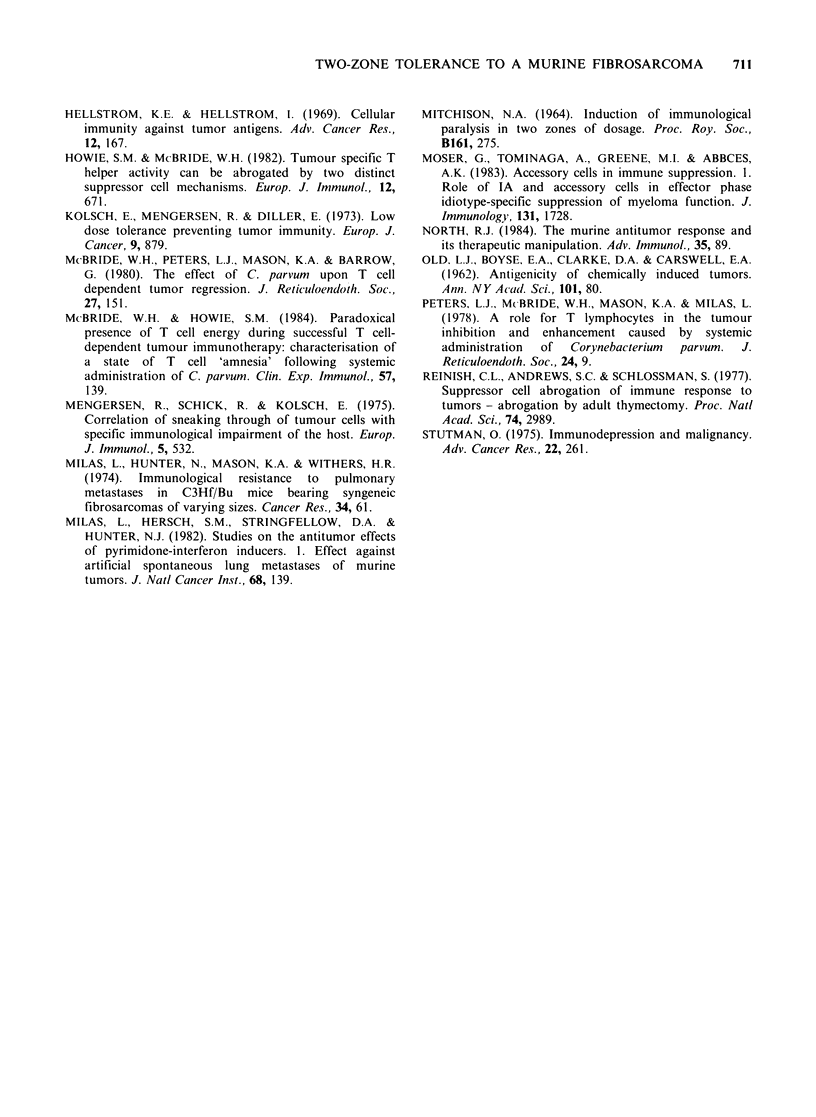# Induction of tolerance to a murine fibrosarcoma in two zones of dosage--the involvement of suppressor cells.

**DOI:** 10.1038/bjc.1986.122

**Published:** 1986-06

**Authors:** W. H. McBride, S. E. Howie

## Abstract

Small size inocula (10(1)-10(3) cells) of cells from a syngeneic methylcholanthrene-induced fibrosarcoma (FSA) induced tolerance when injected s.c. into C3Hf mice. Mice were unable to respond to subsequent challenge with moderate, immunogenic doses of FSA. Tolerance was demonstrated in an in vivo transfer (Winn) assay and an in vitro tumour-specific TH cell assay. Low zone tolerance was associated with the presence of tumour-specific TS cells in the spleen. Moderate size inocula (10(4)-10(6) FSA cells) were immunogenic but larger cell doses (greater than 10(6)) were again tolerogenic. In the high zone, tolerance was associated with both tumour-specific TS cells and non T suppressor cells that were not tumour-specific. These results support the view that immunogenic tumours, as they grow from small cell numbers, might be able to escape host surveillance by specifically tolerizing the immune system. They also suggest that large tumour burdens can interfere with the host's immune response by inducing suppressor cells.


*Br. J. Cancer* (1986), **53**, 707–711

### Induction of tolerance to a murine fibrosarcoma in two zones of dosage – the involvement of suppressor cells

W.H. McBride[1] & S.E.M. Howie[2]

[1]*Department of Radiation Oncology, and Jonsson Comprehensive Cancer Center, UCLA Medical Center, Los Angeles, CA 90024, USA;* [2]*Department of Bacteriology, University of Edinburgh Medical School, Teviot Place, Edinburgh, Scotland, UK.*

**Summary** Small size inocula ($10^1$–$10^3$ cells) of cells from a syngeneic methylcholanthrene-induced fibrosarcoma (FSA) induced tolerance when injected s.c. into C3Hf mice. Mice were unable to respond to subsequent challenge with moderate, immunogenic doses of FSA. Tolerance was demonstrated in an *in vivo* transfer (Winn) assay and an *in vitro* tumour-specific $T_H$ cell assay. Low zone tolerance was associated with the presence of tumour-specific $T_S$ cells in the spleen.

Moderate size inocula ($10^4$–$10^6$ FSA cells) were immunogenic but larger cell doses ($>10^6$) were again tolerogenic. In the high zone, tolerance was associated with both tumour-specific $T_S$ cells and non T suppressor cells that were not tumour-specific.

These results support the view that immunogenic tumours, as they grow from small cell numbers, might be able to escape host surveillance by specifically tolerizing the immune system. They also suggest that large tumour burdens can interfere with the host's immune response by inducing suppressor cells.

An important concept in tumour immunology is that tumours grow only if they can avoid host immune responses. An extension of this concept is that immune responses are selective forces during tumour progression. Numerous mechanisms have been envisaged and investigated by which tumours could escape immune defences. Early research revolved around lack of immunogenicity of tumours, shedding and modulation of cell surface antigens and blocking of tumour reactive lymphocytes by antigen-antibody complexes (Hellstrom & Hellstrom, 1969). More recent studies take into account our knowledge that the immune system is composed of complex interacting and self-regulating networks of cells and soluble factors and have focused on whether antigens on progressor tumours have properties that allow them to avoid protective immunity, for example by preferentially stimulating suppressor cell circuits (Moser *et al.*, 1983; Greene, 1980; Fujimoto *et al.*, 1976; Reinisch *et al.*, 1977; Frost *et al.*, 1982; Kolsch *et al.*, 1973; Mengersen *et al.*, 1975; North, 1984; Haubeck & Kolsch, 1982).

The ability of many tumours to stimulate $T_S$ has been established although the conditions under which they are generated and the extent to which they facilitate growth of primary tumours still requires clarification. In our previous studies with a transplantable murine fibrosarcoma (FSA) we found both tumour-specific $T_S$ and non-tumour-specific non-T suppressor cells in the spleens of tumour-bearing mice in the later stages of tumour growth (Howie & McBride, 1982; McBride & Howie, 1984). The tumour grew initially in the face of developing systemic responses that were demonstrably protective. Concomitant immunity (Milas *et al.*, 1982) and tumour-specific responses could be demonstrated by both *in vitro* (Howie & McBride, 1982; McBride & Howie, 1984) and *in vivo* (Peters *et al.*, 1978; McBride *et al.*, 1980) assays. Suppressor cells did, however, appear to act in time to prevent tumour regression and to facilitate late metastatic spread. The development of $T_S$ cells under such conditions has been described by many and most extensively investigated by North and colleagues (North, 1984). These studies, however, shed little light upon mechanisms operating during the initial stages of primary tumour growth which is presumably the most important period in terms of tumour escape.

In previous experiments we assessed only the response to FSA tumours growing from moderate-size inocula ($4\times10^5$ cells). We subsequently varied the initial tumour load so as to build up a more complete picture of the host-tumour relationship. In this paper we show that both small ($10^1$–$10^3$ cells) and large ($10^7$ cells) size tumour inocula induce $T_S$ cells and tolerance and only moderate size inocula induce immunity. This is therefore analogous to the classic two-zone tolerance phenomenon seen with certain soluble antigens (Mitchison, 1964).

Induction of low zone tolerance by methylcholanthrene-induced cells confirms and extends the findings of Kolsch and coworkers (Kolsch *et al.*, 1973; Mengersen *et al.*, 1975; Haubeck & Kolsch, 1982) in other tumour systems. They have investigated in

Correspondence: W.H. McBride
Received 19 September 1985; and in revised form, 7 March 1986

© The Macmillan Press Ltd., 1986

detail the Balb/c plasmacytoma ADJ-PC-5 and have shown that when irradiated plasmacytoma cells are injected in initially low but exponentially increasing doses, the first immunological reaction is $T_S$ cell activation which can prevent *in vitro* $T_C$ cell generation. This phenomenon could explain how immunogenic tumours grow in the first place. Tumours growing naturally from one or a few cells might, in the early stages of tumour growth, induce tumour-specific $T_S$ which would facilitate escape from immune surveillance. This is a non-mutually exclusive alternative to the more common explanation that the agents used to induce such tumours are generally immunosuppressive (Stutman, 1975). As pointed out by Kolsch, it could also explain the propensity some tumours have for growing just as readily, if not more readily, from low as from moderate cell doses – the phenomenon of 'sneaking through' (Old *et al.*, 1962).

### Materials and methods

### *Mice*

$C_3$Hf/Sed//Kam female mice were used. They were about 12 weeks of age at the start of the experiment.

### *Tumour*

The methylcholanthrene-induced syngeneic fibrosarcoma (FSA) used in these experiments has been described in detail previously (Howie & McBride, 1982; McBride & Howie, 1984; Milas *et al.*, 1982; Peters *et al.*, 1978; McBride *et al.*, 1980). It had been transplanted 7–9 times when used. Tumour cell suspensions were made as described (Howie & McBride, 1982; McBride & Howie, 1984).

### *Experimental design*

The aim of these experiments was to examine the effect of varying doses of FSA upon the immune response. Preliminary experiments established that $4 \times 10^5$ cells s.c. gave a strong response which peaked 7 days after challenge (Howie & McBride, 1982). Less than $10^4$ cells gave no response while greater than $10^6$ cells were also less effective. Varying doses of FSA were injected s.c. into the right flank followed 10 days later by $4 \times 10^5$ cells into the left flank. Spleens were removed one week later and their anti-tumour activity assayed.

### *Winn assay*

T cells were enriched from spleen suspensions by passage over nylon wool columns (Howie & McBride, 1982). Cells were mixed with $2 \times 10^4$ viable FSA and injected s.c. into 40 sites in 20 recipient mice per treatment (Peters *et al.*, 1978; McBride, *et al.*, 1980). Sites were palpated for tumour growth. The day 21 results are reported which is the first day when all control sites were 100% positive. The results represent data from two separate experiments.

### *$T_H$ and $T_S$ cell assay*

These assays have been published (Howie & McBride, 1982; McBride & Howie, 1984). In brief, $2.5 \times 10^6 \, ml^{-1}$ spleen cells from mice primed with trinitrophenylated calf red cells were treated with anti-Thy 1.2 and complement and used as a B cell source in all cultures. Spleen ($10^5$ ml) cells from tumour-bearing mice were treated as described in the text and were the source of primed T cells. Lethally irradiated (50 Gy) TNP-FSA cells ($10^4 \, ml^{-1}$) were the source of antigen. Cultures of admixed T cells, B cells and antigen, with appropriate controls, were established in triplicate. The $T_H$ cells are the limiting factor in these assays. Anti-TNP responses were measured on day 5 by indirect plaquing with TNP-SRBC as antigen.

Putative suppressor cells were added at $10^5$ cells $ml^{-1}$ to cell cultures known to be capable of responding i.e. containing splenic T cells from mice receiving $4 \times 10^5$ FSA s.c. 7 days previously. Specificity or non-specificity of suppression was assayed by examining the ability of the putative suppressors to inhibit responses of T cells taken from TNP-CRBC primed mice with TNP-CRBC as antigen (Howie & McBride, 1982; McBride & Howie, 1984).

The anti-Thy 1.2 used was monoclonal 30:H:12 which was a kind gift of Dr Micklem, Department of Zoology, Edinburgh University.

### Results

We used two assays to measure tumour-specific responses. The first was a Winn assay in which nylon wool, non-adherent spleen cells from mice were mixed with viable tumour cells and injected s.c. into normal recipients (Peters *et al.*, 1978; McBride *et al.*, 1980). Immunity is dependent upon primed $Lyl^+2^-$ cells and is immunologically specific (McBridge & Howie, unpublished). The development of immunity in mice receiving standard inocula of tumour cells was prevented by prior inoculation of either low ($10^1$–$10^3$) or high (greater than $10^6$) doses of the same tumour (Figure 1).

Because the Winn assay is not very well-suited to subpopulation analysis we turned to a sensitive *in vitro* assay for tumour specific $T_H$ cell activity (Howie & McBride, 1982; McBride & Howie, 1984) to analyze this phenomenon further. The kinetics of

**Figure 1** Two zone tolerance to FSA as measured by the Winn assay. Mice were pretreated with varying doses of FSA by injecting them into one flank. Ten days later $4 \times 10^5$ FSA cells were injected into the opposite flank. After a further 7 days, splenic T cells were isolated, admixed with viable FSA cells at a 33:1 ratio and recipient mice were injected with inocula containing $2 \times 10^4$ FSA per site. The percentage of positive sites on day 21 is shown, a time when all control sites receiving FSA alone or normal T cells plus FSA, were positive.

**Figure 2** Two zone tolerance to FSA as measured by the $T_H$ cell assay. Mice were treated in an identical fashion as for **Figure** 1 except the level of tumour-specific T cell help within the spleen cell populations was measured as described in **Materials and methods.** The control 100% value represents the PFC response generated by spleen cells from mice receiving $4 \times 10^5$ FSA cells only (no pretreatment). The effect of pretreatment with various doses of FSA is shown (▲—▲). The possibility that suppressor cells were responsible for the decreased responses by some of the pretreated groups was tested for by mixing spleen cells from these groups with spleen cells from control mice receiving only challenge with FSA (100% group). Suppressor cells were present where there was decreased responsiveness (□—□). Treatment with anti-Thy 1.2 plus complement abolished suppressor cells only in the low zone groups (○—○).

responses demonstrated using this assay have previously been shown to parallel closely those of the Winn assay (Howie & McBride, 1982; McBride & Howie, 1984). The $T_H$ assay relies on the recognition *in vitro* of tumour-specific determinants on irradiated trinitrophenylated tumour cells by $T_H$ cells and presentation of TNP to B cells to generate an anti-TNP response. As can be seen in Figure 2 as few as $10^1$–$10^3$ viable FSA cells prevented the development of tumour-specific $T_H$ cell activity in the spleens of mice subsequently challenged with $4 \times 10^5$ cells. Greater than $10^6$ cells had a similar effect. Between these two zones immunity developed.

In both low and high dosage zones, suppressor cells developed (Figure 2). Spleen cells from these mice could inhibit anti-tumour responses of spleen cells from mice receiving only tumour challenge. Low zone suppressor cells were tumour-specific $T_S$ cells in that they were Thy 1.2 positive (Figure 2) and did not suppress anti-CRBC $T_H$ cell responses (Figure 3). High zone suppressor cells contained tumour-specific and non-tumour-specific cells in that whole spleen cells suppressed anti-CRBC and anti-FSA $T_H$ cell responses (Figure 3) whereas nylon wool-passed cells only suppressed anti-tumour responses (Table I). This is a similar result to that already found in mice bearing large tumour burdens (Howie & McBride, 1982; McBride & Howie, 1984).

**Table I** Suppression of anti-FSA and anti-CRBC responses by T cells from high zone tolerant mice.

| | *Percent suppression* | |
|---|---|---|
| | *FSA response* | *CRBC response* |
| Whole spleen | 98 | 95 |
| Nylon wool nonadherent cells | 96 | 25 |

Nylon wool nonadherent spleen cells and non-separated cells from mice receiving $10^7$ FSA s.c. followed by challenge with $4 \times 10^5$ FSA as in **Figure 1** were tested for their ability to suppress anti-FSA and anti-CRBC responses as in **Figures 2** and **3**.

### Discussion

We have shown that this immunogenic fibrosarcoma can induce two zones of immunological tolerance in normal mice. As few as $10^1$–$10^3$ viable cells can induce low zone tolerance while $10^5$–$10^6$

**Figure 3** The specificity of suppressor cells generated by low and high doses of FSA. The ability of spleen cells from mice pretreated with varying doses of FSA and challenged with $4 \times 10^5$ FSA cells as in **Figure 1** to suppress the generation of TNP-CRBC responses was tested. The 100% value represents responses of separated T and B cell populations from TNP-CRBC primed mice with TNP-CRBC as antigen. Non-specific suppressor cells were present only in the high dose groups.

cells stimulate powerful responses. Larger cell doses induce high zone tolerance which is associated with a more complex and more generalized state of immunosuppression. This last state is probably responsible for the marked loss in immunity when this tumour grows large and may allow metastases to develop (Milas *et al.*, 1974).

Low zone tolerance may account for several aspects of tumour behaviour. One of these is 'sneaking through' (Kolsch *et al.*, 1973; Mengersen *et al.*, 1975; Haubeck & Kolsch, 1982). One would predict that for 'sneaking through' to be explained on this basis there would have to be a dose window where tumour take is inibited by the development of immunity but this manifestation of immunity can be masked by larger tumour cell numbers. Below this window tolerance would be induced. Furthermore to see 'sneaking through', the transplanted tumour must be sufficiently resistant to natural immune mechanisms and sufficiently clonogenic to grow from cell doses that induce tolerance. These requirements would explain why 'sneaking through' is not seen with all tumours and opens up the possibility that low zone $T_S$ cell induction may be a more general phenomenon.

It is possible that tumours, even ones capable of inducing immunity, might have initially escaped the attentions of the host immune system by inducing tolerance. It would be interesting to study the highly immunogenic UV-induced tumours in this regard. It should be noted that, with the possible exception of certain virus-coded products, there is no compelling reason to consider tumour antigens as being anything other than self or minimally altered self components, perhaps exceptional only in the amount and timing of their expression. One might expect responses to such antigens to be under close suppressor cell control. Under natural conditions anti-tumour responses might therefore require breakage of a tolerant state and could be considered as largely autoimmune in nature.

Finally, we previously noted that immunotherapy of this tumour with *C. parvum* was only effective when inocula of moderate size were used (Peters *et al.*, 1978). Not only were small-size inocula not rejected but tumour take was actually enhanced. We can now explain the lack of effect of *C. parvum* on small size inocula as being due to the presence of a tolerant state.

These studies reemphasize the need for extreme care when drawing conclusions from experiments where single doses of transplanted tumours are used and suggest that tolerant mice might be a useful tool for studying the effects of the immune system and immuno-therapy on tumour behaviour.

We would like to thank H.R. Withers and K.A. Mason for their discussion and help and Jan Haas for secretarial assistance. We are also indebted to the Cancer Research Campaign of Great Britain, PHS grant numbers CA-31612 and CA-29644 awarded by the National Cancer Institute, DHHS, and California Institute for Cancer Research grant number C850412 for financial support.

### References

FROST, P., PRETE, P. & KERBEL, R.S. (1982). Abrogation of the in vitro generation of the cytotoxic T cell response to a murine tumor – the role of suppressor cells. *Int. J. Cancer*, **30**, 211.

FUJIMOTO, S., GREENE M.I. & SEHAN, A.H. (1976). Regulation of immune response to tumor antigens – role of suppressor cells. *J. Immunol.*, **116**, 791.

GREENE, M.I. (1980). Cellular and genetic basis of immune reactivity to tumor cells. *Contemp. Topics*, **11**, 81.

HAUBECK, H.D. & KOLSCH, E. (1982). Tumor-specific T suppressor cells induced at early stages of tumorigenesis act on the induction phase of cytotoxic T cells. *Immunology*, **47**, 503.

HELLSTROM, K.E. & HELLSTROM, I. (1969). Cellular immunity against tumor antigens. *Adv. Cancer Res.*, **12,** 167.

HOWIE, S.M. & McBRIDE, W.H. (1982). Tumour specific T helper activity can be abrogated by two distinct suppressor cell mechanisms. *Europ. J. Immunol.*, **12,** 671.

KOLSCH, E., MENGERSEN, R. & DILLER, E. (1973). Low dose tolerance preventing tumor immunity. *Europ. J. Cancer*, **9,** 879.

McBRIDE, W.H., PETERS, L.J., MASON, K.A. & BARROW, G. (1980). The effect of *C. parvum* upon T cell dependent tumor regression. *J. Reticuloendoth. Soc.*, **27,** 151.

McBRIDE, W.H. & HOWIE, S.M. (1984). Paradoxical presence of T cell energy during successful T cell-dependent tumour immunotherapy: characterisation of a state of T cell 'amnesia' following systemic administration of *C. parvum*. *Clin. Exp. Immunol.*, **57,** 139.

MENGERSEN, R., SCHICK, R. & KOLSCH, E. (1975). Correlation of sneaking through of tumour cells with specific immunological impairment of the host. *Europ. J. Immunol.*, **5,** 532.

MILAS, L., HUNTER, N., MASON, K.A. & WITHERS, H.R. (1974). Immunological resistance to pulmonary metastases in C3Hf/Bu mice bearing syngeneic fibrosarcomas of varying sizes. *Cancer Res.*, **34,** 61.

MILAS, L., HERSCH, S.M., STRINGFELLOW, D.A. & HUNTER, N.J. (1982). Studies on the antitumor effects of pyrimidone-interferon inducers. 1. Effect against artificial spontaneous lung metastases of murine tumors. *J. Natl Cancer Inst.*, **68,** 139.

MITCHISON, N.A. (1964). Induction of immunological paralysis in two zones of dosage. *Proc. Roy. Soc.*, **B161,** 275.

MOSER, G., TOMINAGA, A., GREENE, M.I. & ABBCES, A.K. (1983). Accessory cells in immune suppression. 1. Role of IA and accessory cells in effector phase idiotype-specific suppression of myeloma function. *J. Immunology*, **131,** 1728.

NORTH, R.J. (1984). The murine antitumor response and its therapeutic manipulation. *Adv. Immunol.*, **35,** 89.

OLD, L.J., BOYSE, E.A., CLARKE, D.A. & CARSWELL, E.A. (1962). Antigenicity of chemically induced tumors. *Ann. NY Acad. Sci.*, **101,** 80.

PETERS, L.J., McBRIDE, W.H., MASON, K.A. & MILAS, L. (1978). A role for T lymphocytes in the tumour inhibition and enhancement caused by systemic administration of *Corynebacterium parvum*. *J. Reticuloendoth. Soc.*, **24,** 9.

REINISH, C.L., ANDREWS, S.C. & SCHLOSSMAN, S. (1977). Suppressor cell abrogation of immune response to tumors – abrogation by adult thymectomy. *Proc. Natl Acad. Sci.*, **74,** 2989.

STUTMAN, O. (1975). Immunodepression and malignancy. *Adv. Cancer Res.*, **22,** 261.